# Variances in the Expression of mRNAs and miRNAs Related to the Histaminergic System in Endometrioid Endometrial Cancer

**DOI:** 10.3390/biomedicines9111535

**Published:** 2021-10-26

**Authors:** Michał Czerwiński, Anna Bednarska-Czerwińska, Paweł Ordon, Magdalena Gradzik, Marcin Oplawski, Dariusz Boroń, Hanna Zientek, Oskar Ogloszka, Beniamin Oskar Grabarek

**Affiliations:** 1 American Medical Clinic, 40-600 Katowice, Poland; czerwinskaa002@gmail.com; 2 Gyncentrum Fertility Clinic, 40-121 Katowice, Poland; 3 Faculty of Medicine, University of Technology in Katowice, 41-800 Zabrze, Poland; 4 Department of Histology, Cytophysiology and Embryology, Faculty of Medicine, University of Technology in Katowice, 41-800 Zabrze, Poland; pawelordon@outlook.com (P.O.); magdalena.gradzik@onet.pl (M.G.); dariusz@boron.pl (D.B.); lek.hanna.zientek@gmail.com (H.Z.); oskarogloszka@gmail.com (O.O.); bgrabarek7@gmail.com (B.O.G.); 5 Department of Gynecology and Obstetrics with Gynecologic Oncology, Ludwik Rydygier Memorial Specialized Hospital, 31-826 Kraków, Poland; marcin.oplawski@gmail.com; 6 Department of Gynecology and Obstetrics, TOMMED Specjalisci od Zdrowia, 40-662 Katowice, Poland

**Keywords:** endometrioid endometrial cancer, grading, microRNA, histaminergic system, molecular marker

## Abstract

Research has indicated higher concentrations of histamine and polyamine in endometrioid tissue in comparison with healthy tissue. The aim of this study was to evaluate changes in the expression patterns of messenger RNA (mRNAs) and microRNA (miRNAs) related to the histaminergic system in endometrial samples and whole blood in women with endometrioid endometrial cancer. The study group consisted of 30 women with endometrioid endometrial cancer qualified for hysterectomy (G1 well-differentiated, 15 cases; G2 moderately differentiated, 8 cases; and G3 poorly differentiated, 7 cases). The control group included 30 women with no neoplastic changes during routine gynecological examinations. The molecular analysis consisted of the microarray analysis of mRNAs and miRNAs related to the histaminergic system, reverse-transcription quantitative polymerase chain reaction (RTqPCR), and enzyme-linked immunosorbent assay (ELISA). Out of 65 mRNAs connected with the histaminergic system, 10 differentiate the samples of tissue and blood obtained from patients with endometrioid endometrial cancer in comparison with the control group (*p* < 0.05). mRNA histamine receptor 1,3 (*HRH1*, *HRH3*), and solute carrier family 22 member 3 (*SLC23A2*) differentiating samples of endometrioid endometrial cancer independent of either G or control. The highest probability of interaction, based on the target score miRDB, between the selected miRNAs and mRNAs was found for the hybrids hsa-miR-1-3p and endothelin 1 (*END1*), hsa-miR-27a-5β and *SLC23A2*. The selected mRNA and miRNA transcripts seem to be promising for molecularly targeted therapies in the context of endometrioid endometrial cancer.

## 1. Introduction

Endometrioid endometrial cancer (EC) is one of the most common malignant tumors in women in developed countries, and the incidence rate is constantly increasing. In the past 10 years, its frequency has increased by 1% each year and presently stands at 79 cases for every 100,000 women in Europe. The median age at the time of diagnosis is 62 years old. Endometrium endometrial cancer is generally detected at the early stages of advancement and commonly manifests itself as abnormal reproductive organ bleeding [1].

Etiopathogenetically, endometrioid endometrial cancer can be divided into two types [2,3]:

- Type I—endometrial adenocarcinoma (80–90% of all diagnosed cases);
- Type II—non-endometrial adenocarcinoma, which includes serous carcinoma, clear-0 cell carcinoma, non-differentiated tumors, as well as malignant mixed Mullerian tumors (MMMTs) [4].

The severity of the endometrioid endometrial cancer is given in surgical/pathomorphological stages. The current FIGO classification of endometrioid endometrial cancer was introduced in 2009 (Table 1).

A higher risk of endometroid endometrial cancer occurs due to the following factors: obesity and overweight (frequently a component of the metabolic syndrome), nulliparity, infertility (including those caused by polycystic ovarian syndrome), diabetes, early menstruation, late menopause, using hormone replacement therapy, cancers producing estrogens, as well as women of a postmenopausal age taking tamoxifen [5]. Among women, obesity is more strictly related to the development of endometrium endometrial cancer than any other cancer. In addition, along with an increase in BMI the incidence of this type of cancer increases [6].

Histamine is a biogenic amine and it has immunomodulating functions, while also being the principal mediator of severe and acute inflammatory reactions as well as immediate hypersensitivity reactions. Histamine works through four different subtypes of receptors: HRH1, HRH2, HRH3, and HRH4. HRH4 is the last subtype that was discovered and is found mainly in cells of the immune system such as mast cells, basophils, eosinophils, monocytes, dendric cells, T-lymphocytes, and NK-cells [7]. Its expression has been described in various types of cancers [8].

The HRH4 gene undergoes expression in endometrioid endometrial cancer. A higher concentration of histamine and polyamines has been observed in endometrium tissues in comparison with healthy tissues. Patients with tumors exhibiting low or average expression of the HRH4 receptor have a higher survival rate than those with high expression of this receptor [9].

In this case, miRNAs (microRNAs) are a class of short sequences of nucleotide RNA, which play a very important role in many diseases [10].

Here, miRNA plays a key role in numerous intracellular mechanisms such as differentiating stem cells of the hematopoietic system, differentiating cells of the skeletal muscles, exocytosis, apoptosis, regulation of insulin secretion, and embryogenesis, as well as in the process of carcinogenesis and the progression of changes in cancers of various origin in the human body [11,12]. MiRNAs also regulate both suppressor genes and oncogenes. Moreover, miRNAs themselves can fulfill such roles in the process of carcinogenesis [13]. The regulation of the expression of genes takes place on multiple levels. At the present, there is greater significance attributed to the epigenetic regulation of gene expression. It includes the phenomenon of sequence-specific regulation of expression by miRNA molecules. This mechanism is connected with the specific properties of sequences of miRNA composed of several nucleotides (19–23 nt), namely, bonding with target mRNAs. miRNA molecules are used and tested as supplementary molecular markers. Characteristics that contribute to the possibility of using miRNAs as markers of the efficiency of treatment and in the diagnosis of diseases include their stability and high resistance to ribonucleases (RNases), pH changes, and temperature. In addition, the analysis of their expression can be evaluated with the use of basic tools of molecular biology such as RTqPCR reaction or microarray technology. miRNA expression is tissue-specific, although it is also influenced by the type of cell, rate of its metabolism, pathophysiological changes connected with the disease, as well as the activation or silencing of the signal pathways in the cell. It is also necessary to remember the dependencies between disease severity and the profile of miRNA expression, which constitutes a doubtless benefit of using these molecules and molecular indicators [14,15,16].

Four years after initial studies, Hannahan and Weinberg emphasized the role of miRNA in tumor initiation, progression, and invasion as potential oncogenes or tumor suppressors depending on target genes [17]. Another proven role of miRNAs is their influence on the change of cancer-cell drug resistance [18]. Circulating miRNAs have also been proven to play a part in cell–cell communication during immune interactions, and, if secreted by cancer cells, they take part in tumorigenesis in the communicating cells. In recent times, there has been much information about miRNAs with diagnostic and/or prognostic value in gastric, esophageal, pancreatic, breast, prostate and colon cancer, non-small-cell lung carcinoma, and hepatocellular carcinoma [19].

The aim of this study was to evaluate changes in the expression pattern of genes related to the histaminergic system and miRNAs potentially regulating their expression in endometrial samples and whole blood in women with endometrioid endometrial cancer.

## 2. Materials and Methods

The study was performed in accordance with the guidelines of the 2013 Declaration of Helsinki on human experimentation. Data confidentiality and patient anonymity were maintained at all times. Patient-identifying information was deleted before the database was analyzed. It is not possible to identify patients on an individual level either in this article or in the database. Informed consent from all patients was obtained. Approvals of the Bioethical Committee operating at the Regional Medical Chamber in Krakow, no. 185/KBL/OIL/2020 and 186/KBL/OIL/2020, 20 September 2020, were obtained for this study.

### 2.1. Patients

The study group consisted of 30 women with endometrioid endometrial cancer qualified for hysterectomy. The control group included 30 women with no neoplastic changes during routine gynecological examinations. Diagnosis and assignment to the study group (G1-G3) and the control group were performed on the basis of routine histopathological examination after radical hysterectomy. In all cases, surgery was performed, which included the radical removal of the uterus and removal of the pelvic and preaortic lymph nodes. The procedure and histopathological examination were carried out at the Ludwik Rydygier Memorial Specialized Hospital in Cracow, Poland, in the Department of Gynecology and Obstetrics and the Laboratory of Histopathology, respectively.

This study included patients over 45 years of age and after the childbearing period, who were being treated at the Department of Gynecology and Obstetrics with Gynecologic Oncology at the Ludwik Rydygier Memorial Specialized Hospital in Cracow, Poland.

Exclusion criteria from the study group were as follows: endometriosis or adenomyosis, non-endometrioid endometrial cancer, adenocarcinoma with squamous elements, coexisting cervical carcinoma, the use of hormone therapy 24 months before surgery, extreme obesity (BMI > 40), and current or previous history of other types of cancers. The histopathological assessment of endometrial tissue samples allowed for the division of the study group according to the degree of histological differentiation: G1 (well-differentiated), 15 cases; G2 (moderately differentiated), 8 cases; and G3 (poorly differentiated), 7 cases. The clinical and demographic data of patients and controls were included in the Table 2.

### 2.2. Materials

Endometrial cancer samples were obtained for molecular analysis from all women from the control and study group and then stored in Allprotect Tissue Reagent (Qiagen, Valencia, CA, USA Cat No./ID: 76405), while whole blood was extracted into PAXgene RNA kit tubes. All samples were stored at a temperature of −20 °C until molecular analysis was performed. The same samples were used in each type of molecular analysis described in this work.

### 2.3. Extraction of the Total RNA

TRIzol reagent (INvitrogen Life Technologies, Carlsbad, CA, USA, catalog number 15596026) was used to isolate the total ribonucleic acid (RNA) from the tissue samples according to the manufacturer’s protocol.

In turn, commercially available PAXgene Blood RNA kit (Qiagen, Valencia, CA, USA, Cat No./ID: 762174) and PAXgene Blood miRNA kit (Qiagen, Valencia, CA, USA, Cat No./ID: 763134) were used to extract the total RNA from whole blood RNA as per the manufacturer’s protocol.

The RNA extracts were evaluated qualitatively (agarose electrophoresis with ethidium bromide) and quantitatively (RNA concentration (260 nm) and purified (absorbance ratio 260 nm/280 nm) through the analysis of 18S ribosomal RNA (rRNA) and 28S rRNA. Isolated RNAs were qualified for microarray analysis if they had both a clear 18S rRNA and 28S rRNA during the electrophoretic separation and an absorbance ratio value 260 nm/280 nm in the range 1.8–2.0.

### 2.4. The mRNA and miRNA Microarray Analysis

The assessment of the changes in the expression profile of mRNAs related to the histaminergic system was carried out using oligonucleotide microarray microarrays HG-U133A 2_0 (Affymetrix, Santa Clara, CA, USA), the GeneChip™ 3′IVT PLUS Reagent Kit, and GeneChip™ HT 3′IVT PLUS Reagent Kit (ThermoFisher Scientific, Waltham, MA USAm Catalog Number 902416) according to the manufactures’ protocol.

The first stage of the microarray analysis was associated with preparing and add-ing an exogenous poly-A RNA control to the template. The next step was the synthesis of double-stranded cDNA, with the template being the isolated RNA using the GeneChip 30IVT Express Kit. The mixture was incubated for 2 h at 420 °C. Thereafter, 20 μL of Second Strand Master Mix was added to the mixture, followed by incubation for 1 h at 160 °C and then again for 10 min at 650 °C. Then, 30 µL of IVTMaster Mix for cDNA was added and the whole was incubated for 16 h at 40 °C, which allowed the synthesis of biotinylated aRNAs, which were fragmented using a matrix fragmentation buffer for 35 min at 940 °C. The hybridization mixture was prepared with the GeneChip Hybridization Kit, Wash, and Stain Kit. The Affymetrix Gene ArrayScanner 3000 7G and GeneChip^®^Command Console^®^Software were utilized for analysis of the fluorescence intensity.

The names of the probes and their ID numbers (phrase “histaminergic system”; http://www.affymetrix.com/analysis/index.affx; accessed on 15 March 2021) were obtained from the Affymetrix NetAffx™ Analysis Center. The data were analyzed via microarray scanning GeneArray scanner (Agilent Technologies, Santa Clara, CA, USA).

Next, the microarray miRNA profile was determined by using GeneChip miRNA 2.0 Array (Affymetrix) and GeneArray Scanner 3000 7G (Agilent Technologies) according to the manufacture’s protocol.

To determine which of the differentiating miRNAs of the endometrial cancer samples G1–G3 in comparison to the control samples could potentially affect the expression profile of the differentiating mRNAs, the miRDB tool was used (http://mirdb.org/; accessed on 8 April 2021). According to the information from the miRDB database “This is an online database for miRNA target prediction and functional annotations. All the targets in miRDB were predicted by a bioinformatics tool, MirTarget, which was developed by analyzing thousands of miRNA-target interactions from high-throughput sequencing experiments. Common features associated with miRNA binding and target downregulation have been identified and used to predict miRNA targets with machine learning methods” [20].

### 2.5. Reverse-Transcription Quantitative Polymerase Chain Reaction

Reverse-transcription quantitative polymerase chain Reaction (RTqPCR) was performed to validate the microarray data on the same samples used for the microarray. This was completed using the SensiFast™ SYBR No-ROX One-Step Kit (Bioline, London, UK), where β-actin was used as the endogenous control. The volume of the reaction mixture was 50 µL.

The thermal profile of the reaction was as follows: reverse transcription (45 °C, 10 min), activation of the polymerase (95 C, 2 min), 40 cycles including denaturation (95 °C, 5 s), annealing (60 °C, 10 s), and elongation (72 °C, 5 s). The sequence of primers is presented in Table 3. Analysis was performed with an Opticon™ DNA Engine Sequence Detector (MJ Research Inc., Watertown, MA, USA) using the SYBR Green Quantitect RT-PCR Kit (Qiagen, Valencia, CA, USA). Three technical repetitions were performed for each biological replicate.

The expression was performed as a fold change of the gene expression and compared to the control (the 2^−∆∆Ct^ method, also known as a relative method). If the value of fold change (FC) is higher than 1.0, it means, compared to the control culture, that there is an overexpression of genes, whereas if the FC value is lower than 1.0, compared to the control, there is a downregulation of genes.

The specificity of the performed RT PCR reaction was confirmed by the determination of the melting point (Tm) for each amplimer.

### 2.6. ELISA Assay

The last part of this study involved evaluating changes in the expression of HRH1-4 in endometrial cancer samples and whole blood using the double-sandwich ELISA technique and an ELISA Kit. The pre-coated antibody was human HRH1-4 monoclonal antibody. The detecting antibody was polyclonal antibody with biotin labeled. Samples and biotin labeling antibody were added into ELISA plate wells and then washed out with PBS. Next, the avidin–peroxidase conjugates were added to ELISA wells in order. Eventually, the plate was read at 450 nm, and a standard curve was generated to determine the concentration of leptin in the analyzed samples.

To determine the concentration of the analyzed proteins, we used the human histamine H1 receptor, HRH1 ELISA Kit (MyBioSource, Inc. San Diego, CA 92195-3308, USA, Catalog number MBS2602695), human histamine H2 receptor, HRH2 ELISA Kit (MyBioSource, Inc. San Diego, CA 92195-3308, USA, Catalog number MBS265945), human histamine H3 receptor, HRH3 ELISA Kit (MyBioSource, Inc. San Diego, CA 92195-3308, USA, Catalog number MBS450109), and human histamine H4 receptor, HRH4 ELISA Kit (MyBioSource, Inc. San Diego, CA 92195-3308, USA, Catalog number MBS2023167) in accordance with the manufacturer’s recommendation. Three technical repetitions were performed for each biological replicate.

### 2.7. Statistical Analysis

The Transcriptome Analysis Console program (Thermo Fisher Scientific, Waltham, MA, USA) and STATISTICA 13.3 PL software (Statsoft, Cracow, Poland) were used for the statistical analysis. The ANOVA variance analysis test was also completed alongside the post hoc Tukey test (*p* < 0.05). The results of the changes in the expression of mRNA and miRNA were presented as the fold change (FC). The Pearson correlation coefficient was used to determine the agreement of the expression results of the assessed genes determined in endometrioid endometrial tissues compared to the control.

In order to show which miRNAs were engaged in the expression regulation of selected mRNAs, the mirDB database (http://mirdb.org/; accessed on 8 April 2021) was used. All the predicted targets between mRNA and miRNA have target prediction scores between 50–100 calculated on the basis of an target prediction algorithm based on support vector machines (SVMs) and high-throughput training datasets. All the predicted targets have target prediction scores between 50–100. These scores are assigned by the new computational target prediction algorithm. The higher the score, the more confidence we have in this prediction. A predicted target with a prediction score > 80 is most likely to be real; however, if the score is below 60, one needs to be cautious, and it is recommended to have other supporting evidence as well [20].

## 3. Results

### 3.1. The Results of the Microarray Analysis

From among 22,277 mRNAs present on the HG-U133_A2 microarray slide, 65mRNAs are connected with the histaminergic system. The one-way ANOVA variance analysis conducted at the first stage showed that 10 from among the 65 mRNAs differentiate samples of tissues and blood obtained from patients with endometrioid endometrial cancer in comparison with the control (*p* < 0.05). The indicated microarray profile of gene expression connected with the histaminergic system confirmed the same direction of the change in the expression of individual mRNAs, both in the biopsies of the endometrioid endometrial cancer as well as in the control. A correlation between each gene expression in tissue and whole blood was observed (*p* < 0.05; Table 4).

Subsequently, a post hoc Turkey test was conducted, and it was determined which of the genes specifically differentiated samples of the endometrioid endometrial cancer from the control to a given degree of histopathological severity from the control, which are shared by more than one G, and which differentiated the tested samples from the control independent of G. It was observed that two mRNAs specifically differentiate the G1 and G3 samples from the control, while one gene was characteristic for the G3 samples. On the other hand, *HRH1*, *HRH3*, and *SLC23A2* were transcripts that differentiated samples of endometrioid endometrial cancer independent of G from the control. Moreover, these transcripts were common for biopsies and blood samples (Table 5; *p* < 0.05).

### 3.2. The Results of the RTqPCR

**Figure 1:**
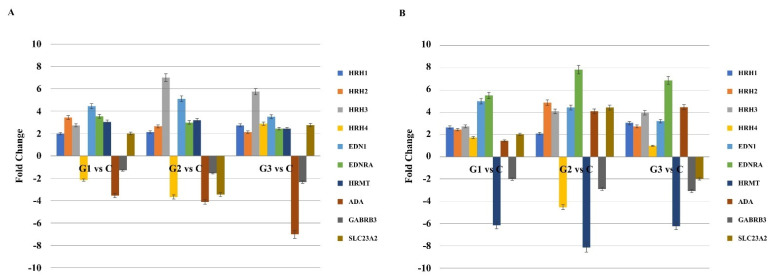
Changes in the expression pattern of genes related to the histaminergic system in endometrial cancer samples (**A**) and whole blood (**B**) in comparison to the control obtained via RTqPCR (*p* < 0.05). (+)—overexpression in comparison to the control; (-)—downregulated in comparison to the control; G—grading; C—control; HNMT—histamine N-methyltransferase; HRH1-3—histamine receptor 1-3; GABRB3—gamma-aminobutyric acid (GABA) A receptor, alpha 3; EDN1—endothelin 1; EDNRA—endothelin receptor type A; SLC223A2—solute carrier family 22 member 3.

Then, the results obtained by the semiquantitative evaluation of gene expression with the use of microarrays were validated by the RTqPCR reaction in samples of endometrial cancer and blood samples. Quantitative RTqPCR was performed to confirm or exclude the pattern of gene expression determined by semi-quantitative microarray analysis. The same direction of change in expression was noted for all transcripts assessed, regardless of the method used. However, the multiple expression change (FC) of the individual genes differed in the two methods (Figure 1; *p* < 0.05).

### 3.3. Expression Pattern of Selected miRNAs

In the following stage, we determined which of the miRNAs differentiating the samples obtained from patients with endometrial cancer from samples obtained from the control group may potentially be involved in the regulations of expression differentiating mRNA using the mirDB database as described in the statistical analysis section. The conducted evaluation showed the strongest connection between *HNMT* and hsa-miR-33a4-5p, *HRH4* and hsa-miR-3915, *EDN1* and hsa-miR-1-30p, as well as hsa-miR-575, *SLC23A2*, and hsa-miR-27a-5p. It was observed that increased expression of *HNMT* and *EDN1* is accompanied by silencing of the expression of the miRNA regulating it. A reverse situation was seen regarding the relation of changes of the expression pattern *HRH4* and *SLC23A2* (Table 6; *p* < 0.05).

### 3.4. The Results of the ELISA

In the final stage of our research, changes in the concentration of HRH1-4 in the samples of tissues of endometrial cancer and blood samples obtained from these patients were evaluated in comparison to the control regarding the level of proteins. In both the biopsies and samples of blood obtained from the tested group, the concentration in all four of the evaluated receptors was higher than that in the control group (*p* < 0.05). Moreover, along with an increase in the histopathological differentiation of endometrial cancer, the expression of the evaluated receptors was higher (C < G1 > G2 > G3; Table 7; *p* < 0.05).

## 4. Discussion

Histamine is one of the best-tested biological molecules in medicine. In the pathogenesis of cancers, this biogenic amine plays an important role, but this mechanism is not fully known and requires further research. Histamine is released from the cells of the immune system, while the biological effect is exerted by interactions with histamine receptors HRH1-4 [7,8]. In the present work, we have seen an increase in the expression of HRH1-3 and a decrease in HRH4 in samples of endometrioid endometrial cancer on the level of mRNA and protein [9].

Modern oncological diagnosis is based on the liquid biopsy method with using liquid biological material, such as blood, plasma, serum, and washings. It has been reported that cancer cells release nucleic acid fragments into the bloodstream and a liquid biopsy can be used [21]. In our study, we tried to check if the expression profile of the assessed genes in endometrial cancer tissue compared to the control is reflected in the blood. If that were the case, these selected markers related to the histaminergic system could be used in predicting the risk of developing endometrial cancer (prophylaxis) or the response to treatment. The obtained results indicated that, in the tissue and blood samples for the given genes, the same direction of change in expression is observed; however, the FC value is at different levels. This may be due to the fact that when using liquid blood as a biological material, the source of nucleic acid is peripheral blood mononuclear cells (PBMCs). It is possible that other factors influencing gene expression than in tissue are released into the blood. Therefore, it would be reasonable to increase the size of the groups or cohort studies. It should also be noted that the greatest correlation between the expression of blood and tissue transcripts occurs when comparing G1 vs. C, where the group was the most numerous. It is therefore advisable to continue research in this area.

Wang et al. showed a statistically significant increase in the transcriptive activity of *HRHS* and *HRH2* with a simultaneous almost-complete silencing of the expression of *HRH3* and *HRH4* (*HRH1 > HRH2 > HRH3 > HRH4*) in the HEC-1 endometrioid adenocarcinoma cell line [22].

The activation of the HRH3 receptor results in a decrease in the cyclical concentration of adenosine monophosphate (cAMP) in the cell cytoplasm, which translates into inhibition of the transduction of signal due to HRH1 and HRH2 receptors. In addition, the HRH3 receptor in influencing the expression of A2 phospholipase, phosphoinositide 3-kinase (PI3K) also inhibits the signaling pathway dependent on IP_3_, which translates into the decrease in the intracellular concentration of calcium ions and the activation of the non-canonical pathway wingless-related integration site (Wnt)/β-catenin. We must also take into account the fact, that the H3 receptor functions as an autoreceptor on the principle of negative feedback, influencing the concentration of histamine and the release of other neurotransmitters [23]. It must also be remembered that the (Wnt)/β-catenin pathway plays an important role in the cells achieving the appropriate endometrium of the phenotype of cancer cells as well as in the promotion of epithelial to mesenchymal transition (EMT) [24,25]. On the other hand, the results we obtained of the expression of HRH3 are in agreement with those described by Jęda et al. [26] and correspond to previously obtained results of the profile of gene expression connected with EMT in endometrioid endometrial cancer [27]. Thus, it seems that HRH3 may play the role of a supplementary marker of the strengthening of endometrioid endometrial cancer and the dedifferentiation of cells (G1 > G2 > G3).

Initial research about the participation of HRH4 in carcinogenesis concerns breast cancer. Its expression was observed both in biopsies as well as in commercially available MDA-231 and MCF-7 cell lines [28,29]. In our research on the levels of both the transcriptome and protein, we have seen a decrease in the expression of HRH4 in endometrioid endometrial cancer in comparison to the control, which is in agreement with the data presented by The Cancer Genome Atlas (TCGA) [30]. Sterle et al. [8] pointed out that HRH4 can be viewed in the context of anti-cancer response in breast cancer [8].

Studies conducted on an animal model of mice with an HRH4 deficiency have shown the key role of this receptor in inflammatory reactions. This is why there are clinical tests being conducted with the aim of verifying the usefulness and safety of using selective HRH4 antagonists in allergic and autoimmune diseases, including asthma, rheumatoid arthritis, and atopic dermatitis. The results show that HRH4 exhibits immunosuppressive activity and impacts the tumor microenvironment. The tests conducted on mice have shown that in the case of mice with breast cancer with an attenuated expression of HRH4, the survival rate of lab mice was higher than that of wild mice [31]. In turn, population-based studies in Denmark have shown a lack of relationship between the risk of developing ovarian cancer and using antihistamine drugs [32]. This information indicates knowledge regarding the histaminergic system and its role in the carcinogenesis process is still insufficient. This is why further research is fully justifiable.

Our research has also shown that the following genes coding proteins participating in the angiogenesis process are connected with the histaminergic system: EDN1 and EDNRA. Endothelin-1 and -2 activate two receptors coupled with proteins G, ETA, and ETB with equal affinity, while endothelin-3 has a weaker affinity to the ETA subtype. Endothelin-1 is characterized by the strongest capabilities of narrowing the blood vessels in the human cardiovascular system, and it works long term [33]. Research that has been carried out until now indicates that EDN1 and its receptor (EDNR) are related to cancer progression. This is why current studies concern the use of EDNR antagonists in the context of carcinogenesis. These studies were based on the hypothesis that the EDN1-EDNR axis activates the MAPK-ERK signal pathway, which is necessary for the survival of cells that have been altered by cancer [34]. In our study, the obtained results allow us to confirm the fact that in the studied biopsies, an overexpression of the EDN1 and EDNRA genes occurred in comparison with the control group, independent of the degree of the histopathological endometrioid endometrial cancer (G). The greatest overexpression in comparison with the control group took place in G2, and it underwent a gradual decrease in the case of G3 samples. In the blood samples, similar results can be observed; however, the highest overexpression of EDN1 was seen in G1 samples (EDNRA, similar to biopsies, had the highest overexpression in G2 in comparison with the control). Studies have proven that the inhibition of the mutated Epidermal Growth Factor (EGF) promotes the release of endothelin-1 (EDN1), which strengthens as cells that make up the mass of the tumor lose the epithelial phenotype in exchange for a mesenchymal one (epithelial–mesenchymal transition), which is a key factor determining metastasis [34]. This is in agreement with our observations made in the present work, as well as in a previous study where the expression of genes connected with EMT in endometrioid endometrial cancer samples was evaluated [27], and it is evidence of the progression of endometrioid endometrial cancer as well as the cells acquiring metastasis potential.

The last gene that differentiated samples of endometrioid endometrial cancer from the control was solute carrier family 23 member 2 (*SLC23A2*). Current studies are focused on the polymorphism of the single nucleotide (SNP) of the *SLC23A2* gene. Two SNPs located in the *SLC23A2* intron region (rs6133175, rs1776948) were connected with the risk of chronic lymphocytic leukemia (CLL) in a clinical control study (*n* = 1.691). However, there was no connection detected between the risk of CLL and the presence of ascorbate in the diet, while the level of ascorbate was not measured. In a Polish cohort study, another SNP (rsl12479919) was inversely correlated with the risk of stomach cancer (*n* = 693) [35]. In our study, the expression of the *SLC23A2* genes in biopsies of endometrioid endometrial cancer varied. In the test group, there was a decrease in the expression of *SLC23A2* in relation to the control group. In the biopsies included among the G1 and G3 groups, overexpression was observed in comparison with the control. On the other hand, in blood samples from the G3 group, the study results showed a lowered expression of the *SLC23A2* gene; however, in the biopsies, it was overexpressed in comparison with the control. The potential reason for the differences may be the relatively small amount of samples in a given G group. Moreover, it seems that the profile of gene expression when the tested material is whole blood is influenced by a greater number of factors, including cytokines regulating the expression of other genes coding cytokines and growth factors. In addition, taking into account the assumptions of liquid biopsy, based on our study it may be assumed that *SLC23A2* is not an ideal candidate for a nuclear marker, which would be useful in the context of endometrioid endometrial cancer [36].

In the last stage of our analysis, we specified the impact of miRNA molecules on the mRNA transcriptome connected with the histaminergic system of endometrioid endometrial cancer.

It was confirmed that the deficiency in the expression of miRNA correlates with the changes of genes, both on the tissue level as well as circulating blood miRNA, which makes miRNA a potential diagnostic or prognostic biomarker in endometrioid endometrial cancer [37]. A detailed analysis of the relationship between ncRNA and endometrial cancer has recently been carried out by Piergientili et al. [38] and Cavaliere et al. [39]. Non-coding RNAs (ncRNAs) are RNA molecules that do not have an open reading frame. In recent years, a large number of non-coding RNAs have been identified that perform various functions, including being responsible for the regulation of other RNAs at the level of transcriptional, post-transcriptional and translational control ubiquitination and protein degradation. Long non-coding RNAs (lncRNAs) constitute a numerous and extremely diverse class of transcripts, which, despite the fact that they do not encode proteins, play an important role in many cellular processes. Recent data confirm that changes in ncRNA expression can be used as prognostic markers in endometrial cancer. This is possible owing to the development of the next-generation sequencing (NGS) technique [38,39].

Moreover, the most significant miRNAs with distorted expression in the endometrioid endometrial cancer were identified: hsa-miR-108, hsa-miR-106a, hsa-miR-181a, hsa-miR-210, hsa-miR-423, hsa-miR-103, hsa-miR-107, hsa-miR-let-7c, hsa-miR-205, hsa-miR-449, hsa-miR-429 (upregulated); hsa-miR-let-7e, hsa-miR-221, hsa-miR-30c, hsa-miR-152, hsa-miR-193, hsa-miR-204, hsa-miR-99b, and hsa-miR-193b (downregulated) [40,41,42]. Nevertheless, it must be taken into account that the studies were conducted on a population of ethnically Asian women. Moreover, these works did not focus on the analysis of miRNAs connected with the histaminergic system, and only a “general” transcriptome was designated for miRNAs whose expression changes in the endometrioid endometrial cancer in comparison with the control.

In the present work, changes in the expression of miRNAs connected with the histaminergic system were observed: *HNMT* (overexpression), *HRH4* (silencing), *EDN1* (overexpression), and *SCL23A2* (silencing), both in tissues as well as in the blood samples collected from patients suffering from endometrioid endometrial cancer in comparison with the control group. These changes correlate to the change in expression of miRNAs: hsa-miR-33a4-5p (silencing), hsa-miR-3915 (overexpression), hsa-miR-1-30p (silencing), hsa-miR-575 (silencing), and hsa-miR-27a-5p (overexpression). From the miRNAs detected in our analysis, most of them were responsible for the regulation of a single studied gene; however, in the case of two miRNAs (hsa-miR-1-30p, hsa-miR-575), they were responsible for the regulation of a single mRNA (*EDN1*). Interestingly, the excessive expression of target mRNAs was connected with the silencing of the expression of the studied miRNAs and vice versa; the silencing of target mRNAs was connected with the overexpression of miRNAs. These changes significantly influence translation. In all of the studied groups, a higher level of *HRH1-4* receptor proteins was observed (the maximum increase was obtained in the G1 group), while in the case of mRNA *HRH4,* we observed downregulation, while the hsa-miR-3915 molecule potentially regulated the expression of this transcript.

Hsa-miR-33a4-5p, which in our analysis underwent downregulation in the studied group of patients in comparison with the control, exhibited similarity to one of the miRNAs studied by Hiroki et al. (miR-33a), exhibiting downregulation in endometrial can and statistically significant (*p* = 0.002) downregulation of a > 2-fold change (6.1), which is in compliance with the downregulation of hsa-miR-33a5p, shown in the present work [43]. In addition, the miR-33a family is a known microRNA responsible for the suppression of the migration and invasion of cancer cells in such cancers as HCC or prostate cancer; therefore, the downregulation of its expression can promote cancer changes [44,45].

Interestingly, hsa-miR-575, which was silenced in our study in patients with EEC, also exhibits downregulation in HPV (+) tonsil tumors, while its expression is not suppressed in HPV (−) tonsil tumors. Moreover, its overexpression was shown in breast cancer cells [46,47,48].

The miR-27a family is a known microRNA group whose representatives may play a role both as oncogenes as well as suppressive agents [48].

Zhou et al. described the overexpression of isoform miR-27a-5p in gastric cancer tissue and cells lines, which complies with our results in that the overexpression of miRNAs was connected with a lowered expression of the *SCL23A2* gene [49]. The miR-27-a miRNAs also show up in the subject literature in the context of mice mast cells, participating in the histaminergic system; their high expression characterizes bone marrow-derived mast cells [50].

The strengths of our study include the evaluation of the changes in the pattern of expression of genes connected with the histaminergic system at the level of the mRNA transcriptome and proteins, as well as indicating the potential role of miRNA in the regulation of selected transcripts. Furthermore, we used modern molecular biology techniques (RTqPCR microarray and ELISA), and we also marked gene expression in biopsies of the endometroid endometrial cancer and in control samples, as well as in the blood of the same patients of the test and control groups.

Of course, beyond the strengths of our study, there are a few limitations to it. Firstly, it would be reasonable to increase the size of the control and study groups as well as patients in the G1–G3 subgroups. A valuable supplement to the research would also be to extend the observation period in order to determine, inter alia, average survival time of patients undergoing hysterectomy. Of course, we must remember that further analysis is required to be performed on a larger population, while SNP should also be studied in the context of the histaminergic system and its connection with the endometrioid endometrial cancer.

## 5. Conclusions

On the basis of the obtained results, a complex structure of the histaminergic system was found. The conducted molecular analysis showed that *HRH1-4*, *EDN1*, *EDNRA*, *SLC23A2*, and *GABRB3* mRNA, as well as miR-33a-5p, miR-3915, miR-1-3p, miR-575, and miR-27a-5p are connected with the histaminergic system in endometrioid endometrial cancer and may be treated as supplementary, diagnostic, molecular markers. The highest probability of interaction, based on the target score miRDB, between the selected miRNAs and mRNAs was found for the hsa-miR-1-3p and *END1* hybrid and the hsa-miR-27a-5β and *SLC23A2* hybrid. The selected mRNA and miRNA transcripts seem to be promising goals for molecularly targeted therapies in the context of endometrioid endometrial cancer. It also seems that these results may be used in the evaluation of the severity of endometrioid endometrial cancer. However, further analysis is necessary.

## Figures and Tables

**Table 1:** System of endometrioid endometrial cancer classification according to FIGO from 2009.

I	Tumor Strictly Limited to the Body of the Uterus
IA	No invasion or myometrial invasion <50%
IB	Myometrial invasion ≥50%
II	Cervical stromal invasion, but not beyond the uterus
III	Local and/or regional invasion
IIIA	Tumor involves serosa and/or adnexa
IIIB	Vaginal and/or parametrial involvement
IIIC	Pelvic and/or para-aortic lymph node involvement
IIIC1	Pelvic lymph node involvement
IIIC2	Cancer has spread to the para-aortic lymph nodes, with or without spread to the regional pelvic lymph nodes
IV	Involvement of bladder and/or rectal mucosa and/or distant metastasis
IVA	Involvement of bladder and/or rectal mucosa
IVB	Distant metastases including abdominal and/or inguinal lymph node metastases

**Table 2:** Characteristic of patients included in a control group and a study group.

	C (*n* = 30 Cases)	G1 (*n* = 15 Cases)	G2 (*n* = 8 Cases)	G3 (*n* = 7 Cases)
Age (years)	65.36 ± 10.29	67.22 ± 8.04	68.4 ± 10.09	64.88 ± 12.02
Height (m)	1.63 ± 0.14	1.59 ± 0.08	1.62 ± 0.05	1.59 ± 0.04
Weight (kg)	72.99 ± 13.95	74.41 ± 11.79	85.77 ± 21.99	85.22 ± 13.11
BMI	28.77 ± 7.14—overweight	29.01 ± 3.14 overweight	36.15 ± 10.44—1st degree of obesity	33.18 ± 5.4—1st degree of obesity
Hypertension	6 (20%)	9 (60%)	5 (62.5%)	5 (71.4%)
Diabetes I	3 (10%)	4 (26.67%)	2 (25%)	2 (28.7%)
Diabetes II	6 (20%)	6 (40%)	3 (37.5%)	3 (42.86%)
Hypertension + Diabetes I	1 (3.33%)	1 (6.67%)	1 (12.55)	1 (14.29%)
Hypertension + Diabetes II	2 (6.67%)	2 (13.33%)	1 (12.5%)	1 (14.29%)

C—control; G—grading; BMI—Body Mass Index; average ± standard deviation.

**Table 3:** The sequence of primers used in RTqPCR.

mRNA	Nucleotide Sequence	
*HRH1*	Left	TATGTTTAAGTGGTTATTGGGTTGT
	Right	ACCAAAACTCAAATCTTAATACAAT
*HRH2*	Left	ATAGTTTTGGTTTTAGTTTTGTTGT
	Right	CAAAACATATTCATATCCCTTCACT
*HRH3*	Left	TTTTATATTGGGTTTAGTAGGGTGATAT
	Right	CTCCCAACTCAAAATAACTAATCCA
*HRH4*	Left	TTAGTATTTTGGGAGGTTAAGGTG
	Right	TTCTATTACCAAACTAACAAACTCC
*HNMT*	Left	GTTTGTAGTTAAGATATTGAATTTTGA
	Right	AAAATCATCCTAAAAAAAACATA
*EDN1*	Left	AGGTTTGAAATTTTGTATTTTTTTT
	Right	CCACCTTACTAAAACTACCCCTACA
*EDNRA*	Left	ATAAATGTATGAGGAATGGTTTTAA
	Right	AAAAAAAACAACTTACAAAAAAATAC
	Right	CAATAAAATCCCACAAAAATCAAT
*GABRB3*	Left	TTGTATGGGTTTAGAATTATTATGA
	Right	ATACTCCACAATAAAAAACTACAAA
*SLC23A2*	Left	GTGTGGGTAAAGGGAATAAATTATT
	Right	ACCACAAAACACAACAAAAACTATC

HNMT—histamine N-methyltransferase; HRH1-3—histamine receptor 1-3; GABRB3—gamma-aminobutyric acid (GABA) A receptor, alpha 3; EDN1—endothelin 1; EDNRA—endothelin receptor type A; SLC223A2—solute carrier family 22 member 3; ADA—adenosine deaminase.

**Table 4:** Microarray profile of the expression of genes connected with the histaminergic system in G1–G3 samples of endometrioid endometrial cancer in comparison with the control.

mRNA	Tissue	Whole Blood	Pearson Correlation Coefficient	Tissue	Whole Blood	Pearson Correlation Coefficient	Tissue	Whole Blood	Pearson Correlation Coefficient
	G1 vs. C			G2 vs. C			G3 vs. C		
*HRH1*	+1.98	+2.65	0.82	+2.14	+2.11	0.61	+2.75	+3.04	−0.54
*HRH2*	+3.11	+2.45	0.96	+3.02	+4.88	−0.17	+2.14	+2.74	0.51
*HRH3*	+2.74	+2.74	−0.97	+6.14	+4.10	0.36	+5.75	+3.96	−0.32
*HRH4*	−2.14	−1.55	−0.84	−3.66	−4.48	−0.15	−2.88	−1.22	−0.31
*EDN1*	+4.45	+5.01	−0.87	+5.11	+4.44	0.27	+4.01	+3.22	0.80 *
*EDNRA*	+3.66	+7.14	−0.95	+3.74	+8.01	−0.61	+2.44	+6.88	−0.86 *
*ADA*	−3.54	−6.14	0.84	−3.88	−8.14	−0.32	−7.01	−6.22	−0.34
*HNMT*	+3.05	+1.45	0.69	+3.19	+5.08	−0.70	+2.44	+4.47	−0.56
*GABRB3*	−1.29	−2.01	0.42	−1.54	−2.88	−0.10	−2.33	−3.05	−0.17
*SLC23A2*	+2.01	+1.99	0.78	−3.45	+4.44	−0.27	+2.77	−1.98	−0.24

(+)—overexpression in comparison to the control; (−)—downregulated in comparison to the control; G—grading; C—control; HNMT—histamine N-methyltransferase; HRH1-3—histamine receptor 1-3; GABRB3—gamma-aminobutyric acid (GABA) A receptor, alpha 3; EDN1—endothelin 1; EDNRA—endothelin receptor type A; SLC223A2—solute carrier family 22 member 3; ADA—adenosine deaminase; * statistically significant relationship between gene expression in tissue and whole blood (*p* < 0.05).

**Table 5:** Transcripts of genes associated with the histaminergic system, differentiating the tissue and whole blood samples obtained from patients with endometrial cancer in comparison to the control group (*p* < 0.05).

	Compared Group	mRNA
Tissue samples	G1 vs. C	*HRH2*, *HRH4*
	G2 vs. C	*HNMT*
	G3 vs. C	*EDN1*, *EDNRA*
	G1, G2 vs. C	*GABRB3*
	G1, G3 vs. C	*-*
	G2, G3 vs. C	*ADA*
	G1, G2, G3 vs. C	*HRH1*, *HRH3*, *SLC23A2*
Whole blood samples	G1 vs. C	*HRH2*, *HRH4*
	G2 vs. C	*EDNRA*
	G3 vs. C	*HNMT*, *EDN1*
	G1, G2 vs. C	*ADA*
	G1, G3 vs. C	*-*
	G2, G3 vs. C	*GABRB3*
	G1, G2, G3 vs. C	*HRH1*, *HRH4*, *SLC23A2A*

G—grading; C—control; HNMT—histamine N-methyltransferase; HRH1-3—histamine receptor 1-3; GABRB3—gamma-aminobutyric acid (GABA) A receptor, alpha 3; EDN1—endothelin 1; EDNRA—endothelin receptor type A; SLC223A2—solute carrier family 22 member 3; ADA—adenosine deaminase.

**Table 6:** Expression profile of miR-33a-5p, miR-3915, miR-1-3p, miR-575, and miR-27a-5p in endometrial tissue and whole blood of patients with endometrial cancer, compared to the control.

mRNA	miRNA	Target Score mRNA:miRNA	Tissue			Whole Blood		
			miRNA			miRNA		
			G1 vs. C	G2 vs. C	G3 vs. C	G1 vs. C	G2 vs. C	G3 vs. C
*HNMT*	hsa-miR-33a-5p	54	+2.74 *	−3.41 *	−2.41 *	−2.04 *	−2.55 *	−4.01 *
HRH4	hsa-miR-3915	53	+2.01 *	+2.77 *	+3.01 *	+3.44 *	+2.55 *	+3.77 *
EDN1	hsa-miR-1-3p	94	−2.45 *	−3.44 *	−1.44	−2.01 *	−3.11 *	−2.06 *
	hsa-miR-575	70	−2.98 *	−3.14 *	−1.98 *	−2.41 *	−2.98 *	−2.01 *
*SLC23A2*	hsa-miR-27a-5p	91	+2.44 *	+2.07 *	+1.55 *	+2.01 *	+1.44	−1.05

(+)—overexpression in comparison to the control; (−)—downregulated in comparison to the control; G—grading; C—control; miRNA—micro RNA; HNMT—histamine N-methyltransferase; HRH1-3—histamine receptor 1-3; EDN1—endothelin 1; SLC223A2—solute carrier family 22 member 3; *—vs. C; *p* < 0.05.

**Table 7:** Differences in concentration of HRH1, HRH2, HRH3, and HRH4 in endometrial cancer samples and whole blood obtained from patients with endometrioid endometrial cancer and the control (ELISA assay).

	Tissue (ng/mL)				Whole Blood (ng/mL)			
	C	G1	G2	G3	C	G1	G2	G3
HRH1	4.44 ± 0.48	9.58 ± 1.22 *	10.44 ± 0.94 *	16.74 ± 1.07 *	3.18 ± 0.48 *	9.18 ± 1.17 *	12.47 ± 0.82 *	16.11 ± 1.44 *
HRH2	1.47 ± 0.26	2.44 ± 0.55 *	4.66 ± 1.04 *	8.14 ± 1.58 *	1.14 ± 0.96 *	2.88 ± 0.54 *	4.25 ± 0.47 *	9.03 ± 1.18 *
HRH3	2.18 ± 0.73	3.44 ± 0.26 *	4.69 ± 0.37 *	7.11 ± 0.12 *	2.14 ± 0.99 *	2.96 ± 0.1.04 *	5.55 ± 0.63 *	8.03 ± 1.09 *
HRH4	9.12 ± 0.25	7.64 ± 0.49 *	3.14 ± 0.19 *	1.01 ± 0.34 *	10.54 ± 0.25 *	5.24 ± 0.12 *	2.14 ± 0.77 *	1.11 ± 0.24 *

G—grading; C—control; HRH1-4—histamine receptor 1-4; mean ± standard deviation; *—vs. C; *p* < 0.05.

## Data Availability

The data used to support the findings of this study are included in the article. The data will not be shared because of third-party rights and commercial confidentiality.
